# The Mre11 protein interacts with both Rad50 and the HerA bipolar helicase and is recruited to DNA following gamma irradiation in the archaeon *Sulfolobus acidocaldarius*

**DOI:** 10.1186/1471-2199-9-25

**Published:** 2008-02-22

**Authors:** Achim Quaiser, Florence Constantinesco, Malcolm F White, Patrick Forterre, Christiane Elie

**Affiliations:** 1 Univ Paris-Sud, Institut de Génétique et Microbiologie, CNRS UMR 8621, Bâtiment 409, F 91405 Orsay Cedex, France; 2 Centre for Biomolecular Sciences, University of St Andrews, North Haugh St Andrews, Fife KY16 9ST, UK

## Abstract

**Background:**

The ubiquitous Rad50 and Mre11 proteins play a key role in many processes involved in the maintenance of genome integrity in Bacteria and Eucarya, but their function in the Archaea is presently unknown. We showed previously that in most hyperthermophilic archaea, *rad50-mre11 *genes are linked to *nurA *encoding both a single-strand endonuclease and a 5' to 3' exonuclease, and *herA*, encoding a bipolar DNA helicase which suggests the involvement of the four proteins in common molecular pathway(s). Since genetic tools for hyperthermophilic archaea are just emerging, we utilized immuno-detection approaches to get the first *in vivo *data on the role(s) of these proteins in the hyperthermophilic crenarchaeon *Sulfolobus acidocaldarius*.

**Results:**

We first showed that *S. acidocaldarius *can repair DNA damage induced by high doses of gamma rays, and we performed a time course analysis of the total levels and sub-cellular partitioning of Rad50, Mre11, HerA and NurA along with the RadA recombinase in both control and irradiated cells. We found that during the exponential phase, all proteins are synthesized and display constant levels, but that all of them exhibit a different sub-cellular partitioning. Following gamma irradiation, both Mre11 and RadA are immediately recruited to DNA and remain DNA-bound in the course of DNA repair. Furthermore, we show by immuno-precipitation assays that Rad50, Mre11 and the HerA helicase interact altogether.

**Conclusion:**

Our analyses strongly support that in *Sulfolobus acidocaldarius*, the Mre11 protein and the RadA recombinase might play an active role in the repair of DNA damage introduced by gamma rays and/or may act as DNA damage sensors. Moreover, our results demonstrate the functional interaction between Mre11, Rad50 and the HerA helicase and suggest that each protein play different roles when acting on its own or in association with its partners. This report provides the first *in vivo *evidence supporting the implication of the Mre11 protein in DNA repair processes in the Archaea and showing its interaction with both Rad50 and the HerA bipolar helicase. Further studies on the functional interactions between these proteins, the NurA nuclease and the RadA recombinase, will allow us to define their roles and mechanism of action.

## Background

The ability to signal and repair DNA damage is essential to any cell and requires many pathways. Among these pathways, recombination processes constitute an important set of systems acting in the repair of DNA breaks and of stalled/collapsed replication forks [1-3]. Besides the ubiquitous recombinase (RecA in Bacteria, Rad51 in Eucarya and RadA in Archaea), the highly conserved Rad50 and Mre11 proteins must play important roles in these processes even if their precise function is still unclear. In Bacteria, these proteins, known as SbcC and SbcD respectively, are involved in the elimination of palindromes in the course of DNA replication and in the repair of double strand breaks (DSBs), inter-strand DNA cross links and collapsed replication forks [4-6]. In Eucarya, Rad50 and Mre11 proteins are associated with a third eucaryal-specific partner, Xrs2 in yeast, Nbs1 in human, and play a key role in a surprising large range of pathways: the repair of DSBs by homologous recombination and, at least in *Saccharomyces cerevisiae*, non-homologous-end-joining, the repair of collapsed replication forks, DNA damage cell checkpoint, the maintenance of telomeres, and the generation (except for *Saccharomyces pombe*) as well as the resection of meiotic DSBs [7,8]. In Archaea, Rad50 and Mre11 homologs have been found in all species [9] and characterization of recombinant proteins from the hyperthermophilic euryarchaeon *Pyrococcus furiosus *showed that they form a tight complex exhibiting activities similar to their bacterial and eucaryal counterparts [10-13]. However, the role of these proteins *in vivo *is presently unknown.

In Eucarya, Rad50 and Mre11 proteins were shown to act at the initiation step of homologous recombination in the resection of broken DNA ends in 3' DNA tails required for recombinase loading and strand exchange, but their precise role in this process is still unclear [14]. In Bacteria, this step is primarily performed via the RecBCD and the RecQ/RecFOR/RecJ pathways [15-17]. In both cases, initiation processes are well understood and emphasize the implication of helicases and 5' to 3' nucleases. The Rad50 and Mre11 proteins form a tight complex that exhibits single-strand endonuclease and 3'-5' exonuclease activities relevant to the phosphoesterase Mre11 [18] together with a mechanical function inherent to Rad50. This protein, related to SMC proteins (for Structural Maintenance of Chromosomes), might be involved in the tethering of broken DNA molecules [19,20]. However, the activities associated with the Rad50-Mre11 complex even in the presence of Xrs2/Nbs1, do not explain how DNA ends are processed into 3' overhangs, suggesting the involvement of additional partners [21].

We found previously that in most hyperthermophilic archaea, *rad50-mre11 *genes are clustered with two unknown genes that we called *nurA *and *herA *and that the four genes are co-transcribed in the crenarchaeon *Sulfolobus acidocaldarius *[22,23]. We characterized recombinant proteins from *S. acidocaldarius *and showed that NurA defines a new nuclease family exhibiting both a single-strand endonuclease activity and a 5' to 3' exonuclease activity on single and double-strand DNA [22], and that HerA is the prototype of a novel DNA helicase family and exhibits the striking property to unwind the DNA helix from both a 3' and a 5' single-strand overhang (bipolar helicase that corresponds to the Mla ATPase described concomitantly by Manzan *et al*. [23,24]. The activities associated with NurA and HerA together with the synteny of *rad50*, *mre11*, *nurA *and *herA *genes, suggest that the four proteins might play a central role in the resection of DNA breaks at the initiation step of homologous recombination in archaea. Indeed, the RecBCD complex, for which no homolog is found in archaea, acts by two helicases of opposite polarity (the 3' to 5' RecB helicase and the 5' to 3' RecD helicase) that bind to DNA ends of each DNA strand and increase the processivity of the entire complex [25,26]. The bipolar helicase HerA might play such a role except that both helicase activities reside in the same protein.

Many hyperthermophilic archaeal species are highly resistant to gamma rays and repair fragmented chromosomes efficiently using pathways that have not been clearly established and that appear to be mostly constitutive [27-30]. As powerful genetic tools for hyperthermophilic archaea are just emerging, we took an immuno-detection approach to investigate the potential roles of the Rad50, Mre11, NurA and HerA proteins in post-irradiation DNA repair in *S. acidocaldarius*. We analyzed the levels and sub-cellular partitioning of Rad50, Mre11, NurA and HerA along with the RadA recombinase in mock-treated and irradiated cells and we checked for the interactions between this set of proteins. Our main results show that 1) all proteins are synthesized in exponentially growing cultures and show constant levels, 2) all proteins display different sub-cellular partitioning 3) Rad50, Mre11 and HerA interact altogether in a constitutive way and 4) after gamma irradiation, both Mre11 and RadA are recruited immediately to chromosomal DNA and remain DNA bound in the course of DNA repair. These data provide the first evidence supporting the involvement of Mre11 in DNA repair processes in the Archaea and showing the interaction between Rad50, Mre11 and the HerA bipolar DNA helicase.

## Results

### Irradiation of *S. acidocaldarius *cells, chromosome fragmentation and subsequent DNA repair

**Figure 1:**
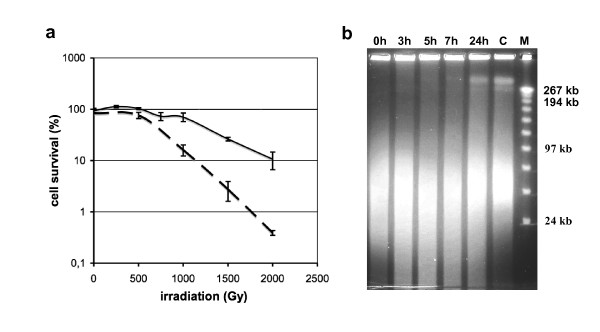
**(a) Survival rates of *S. acidocaldarius *to different doses of gamma rays**. For each dose, irradiations were performed under aerobic (broken line) or limited-aerobic (continuous line) conditions. **(b) Time course analysis of *S. acidocaldarius *chromosomal DNA following a 1000 Gy irradiation**. DNA content of 10^8 ^cells samples, collected after 0, 3, 7 and 24 h of post-irradiation growth, were analyzed by pulsed-field gel electrophoresis. C: DNA from mock treated cells collected at time 0. M: MidRange II PFG molecular weight marker (NEB).

We first determined the dose of gamma irradiation that does not lead to massive cell death but induces chromosome fragmentation and subsequent repair. *S. acidocaldarius *was previously shown to be as radiosensitive as *E. coli *under aerobic conditions [31]. As oxygen is known to dramatically increase radio-sensitivity [32], we compared the survival rates of *S. acidocaldarius *irradiated under aerobic or limited-aerobic conditions. As shown in Fig 1a, the radioresistance is higher under limited-aerobic conditions, with more than 70 % of cells alive after exposure to 1000 Gray of radiation. In order to verify that the 1000 Gray irradiation has induced DNA fragmentation and to determine the timing of repair, we performed a time course analysis of the DNA content of the irradiated cells. Immediately after the irradiation, cells were re-inoculated into fresh medium and further grown up to the stationary phase. Aliquots of post-irradiated cultures were harvested after different times (0, 3, 5, 7 and 24 h) and chromosomal DNA was analyzed by pulsed-field gel electrophoresis (PFGE). Whereas the survival rate determined by cells plating immediately after the 1000 Gray irradiation and corresponding to time 0 is about 70 %, chromosomal DNA analysis shows that irradiated cells have sustained high levels of chromosome fragmentation (Fig 1b, time 0). This indicates that a majority of the damaged cells has eventually recovered and resumed growth. Furthermore, the time course analysis shows that chromosomal DNA was restored in 24 h.

### Analyses of Rad50, Mre11, HerA, NurA and RadA protein levels and partitioning

Concomitantly to DNA analysis, we performed a time course study (from 0 to 24 h) of the amount and the partitioning of each protein between chromosomal-enriched and soluble fractions both in non-irradiated and 1000 Gray irradiated cells. Cell fractionations were performed by gentle lysis with 0.5 % Triton X-100 and low-speed centrifugation through sorbitol allowing the recovery of more than 90 % of total DNA in pellet fractions as shown in the next section. Total, pellet and supernatant fractions from three independent experiments were analyzed in triplicate by Western blotting with specific antibodies raised against each protein, and control cultures were submitted to exactly the same procedure without irradiation in order to rule out the effects of cell manipulation. Loading controls were performed using antibodies raised against *S. acidocaldarius *DNA ligase for which no variation was previously detected (A. Quaiser *et al*., unpublished results). As the DNA-bound portion of the ligase is very weak, we also utilized this protein as an internal control of cell lysis and fractionation. Furthermore, the reliability of these studies was ensured by the immunodetection of four proteins together with the DNA ligase on the same blot.

#### Rad50, Mre11, HerA, NurA and RadA proteins display different sub-cellular partitioning

**Figure 2:**
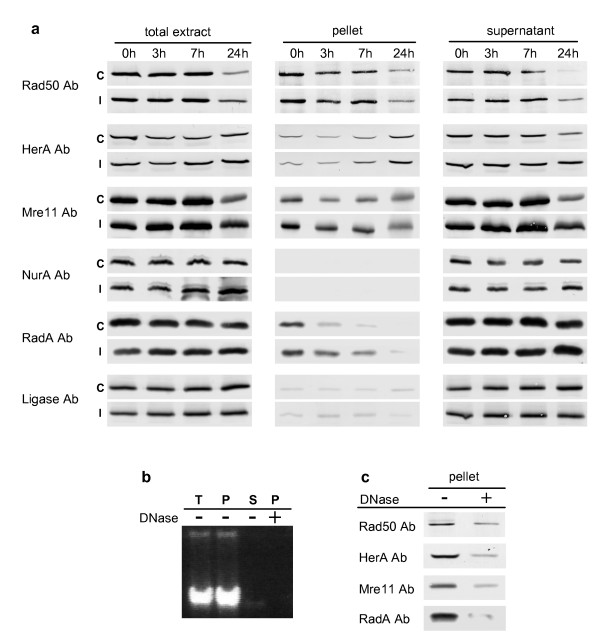
**(a) Analysis of the levels and sub-cellular partitioning of Rad50, Mre11, HerA, NurA and RadA.** Immunoblots were performed on total, chromosomal-enriched and soluble fractions recovered after 0, 3, 7 and 24 h of post-treatment growth. C: non-irradiated *S. acidocaldarius *cells. I: *S. acidocaldarius *cells exposed to a 1000 Gy irradiation under limited-aerobic conditions. DNA ligase was used as a control of cell fractionation and samples loading. **(b) Analysis by gel electrophoresis (1% agarose) of the DNA content of total extract (T), pellet (P), supernatant (S) and pellet from DNase I treated total extract (P +DNase). (c) Western blot analysis of pellets from un-treated (-) and DNase I treated (+) total extracts.**

**Figure 3:**
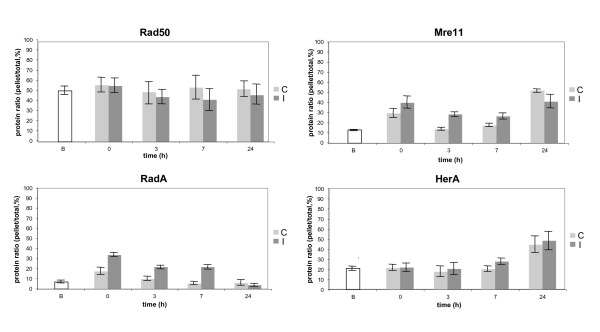
**Statistic analysis of the DNA-bound portion of Rad50, Mre11, RadA and HerA proteins in control (C) and irradiated cells (I)**. Quantifications were performed using the ImageJ 1.38 software [47] from three independent experiments. B: basal ratios determined from cultures (time 0) before any cell manipulation (concentration and ice cooling performed for control and irradiated cells).

We first examined protein patterns in non-irradiated cells (Fig 2a, lanes C). All proteins showed a constant level up to 7 h of growth (lanes C, total extracts). In cells from stationary phase cultures (24 h), the amount of Rad50 and Mre11 proteins decreased significantly whereas HerA, NurA and RadA protein levels remained in the same range. With the exception of the NurA protein, which was only detected in supernatant, a significant portion of all other proteins were recovered in pellet fractions. In order to confirm that these proteins were bound to DNA, cell fractionation was applied on total extract (3 h) treated with DNase I. As shown in Figures 2b and 2c, the DNase I treatment led to the disappearance of nearly all DNA in pellet fractions and to a dramatic decrease of the amount of Rad50, HerA, Mre11 and RadA. Since we performed cell fractionation without any DNA-protein fixation, this indicates a strong association with the chromosome. A statistic analysis of the DNA-bound portion of the four proteins is shown in figure 3. The protein that exhibited the highest percentage of DNA-bound molecules was Rad50 (50 % compared to 20 % for Mre11) showing that this SMC-like protein is associated with DNA constitutively. In the case of HerA, the chromosomal-associated fraction increased along the time course, reaching about 50 % at 24 h, suggesting a progressive recruitment of HerA to DNA from the early exponential phase up to the stationary phase. The DNA-bound portion of RadA is less than 10 % except at time 0 for which a higher amount was observed. Analysis of RadA DNA-bound fraction before cell manipulation indicated that growth arrest and cold shock induces this recruitment to DNA (Fig. 3). This phenomenon is also observed for the Mre11 protein.

#### The Mre11 protein and the RadA recombinase are recruited to chromosomal DNA following gamma irradiation

Comparative analyses of protein patterns from mock treated and irradiated cells (Fig. 2a, comparison between lanes I and C and Fig. 3) show that concomitantly to the irradiation (time 0) and up to seven hours of post-irradiation growth, the DNA bound fractions of both Mre11 and RadA increased significantly, indicating that both proteins were recruited to DNA which suggests their involvement in post-irradiation DNA repair. In total extracts, we observed a slight but reproducible increase of HerA levels at 7 h and 24 h of post-irradiation growth, suggesting that HerA synthesis might be slightly up-regulated. We also noticed that at 24 h, the amount of both Rad50 and Mre11 proteins in total extracts of irradiated cells showed a smaller decrease compared to the decrease observed in control cells, and that this phenomenon was also observed in soluble fractions. This difference might indicate that some un-repaired DNA lesions lead to the maintenance of Rad50 and Mre11 synthesis or to their protection against protein degradation processes.

### The HerA bipolar helicase interacts with the Rad50-Mre11 complex both *in vivo *and *in vitro*

**Figure 4:**
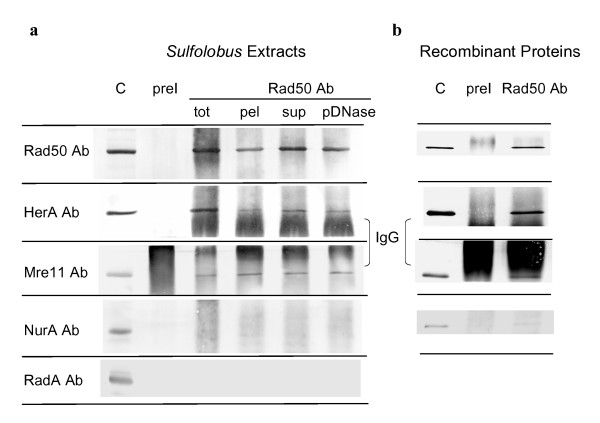
**Co-immunoprecipitation of Rad50, Mre11 and HerA by anti-Rad50 antibodies**. (a) Western blot analysis of immuno-precipitates performed on extracts from exponentially growing *S. acidocaldarius *cells: tot, total extract; pel, chromosomal-enriched fraction; sup, soluble fraction; pDNase, chromosomal-enriched fraction previously treated with DNase I. (b) Western blot analysis of immuno-precipitates performed on a mixture of Rad50, Mre11, HerA and NurA recombinant proteins. C: Western blot control on (a) *S. acidocaldarius *total extracts and (b) recombinant proteins mixtures. preI: immunoprecipitations with pre-immune antibodies.

In order to check potential interactions between Rad50, Mre11, HerA, NurA and RadA proteins, we performed immuno-precipitation assays using total extracts of exponentially growing *Sulfolobus *cells. As shown in Fig. 4a, both Mre11 and HerA were co-precipitated together with Rad50 by anti-Rad50 antibodies, whereas neither NurA nor RadA were recovered. The same result was obtained using either post-irradiated or non-irradiated cell extracts indicating that Rad50, Mre11 and HerA dependent complexes are formed constitutively in *S. acidocaldarius*. Immuno-precipitation assays were also performed using soluble fractions and chromosomal-enriched fractions previously treated with DNase I. In both cases, the Rad50, Mre11 and HerA proteins were recovered indicating that DNA is not required for the formation of these complexes. In order to determine if the interaction between Rad50, Mre11 and HerA required other protein partners, immuno-precipitation assays were performed on a mixture of purified Rad50, Mre11, HerA and NurA recombinant proteins. As shown in Fig. 4b, Rad50, Mre11 and HerA proteins were co-precipitated by anti-Rad50 antibodies indicating a direct interaction between the three proteins.

## Discussion

In this report, we first showed that Rad50, Mre11, NurA, HerA and the RadA recombinase are synthesized in exponentially growing *S. acidocaldarius *cells and display constant levels. Whereas all proteins exhibit different sub-cellular partitioning, we detected soluble protein complexes including Rad50, Mre11 and the HerA bipolar helicase. These results indicate that each protein might play specific roles in addition to its function when combined with its partners. Indeed, the SMC-like Rad50 protein shows the highest level of chromosomal-bound fraction suggesting a constitutive role on DNA. Concomitantly to gamma irradiation and chromosome fragmentation, we observed that a significant portion of Mre11 protein is recruited to DNA (or to Rad50 DNA-bound proteins) and is still DNA-bound in the course of DNA repair. This result strongly suggests that the Mre11 nuclease is actively involved in DNA repair processes and/or act as a DNA damage sensor. As previously described for other archaeal species [30,33], gamma irradiation does not lead to the increase of RadA levels. However, RadA is immediately recruited to DNA which indicates that DNA damaged products exhibiting single-strand portions such as 3' single-strand overhangs, gapped DNA or stalled replication forks might be recognized by the recombinase and/or that RadA also acts as a DNA damage sensor. For both RadA and Mre11 proteins, the recruitment to DNA is also observed to a weaker extent in control cells concomitantly to cold shock, growth arrest and consequently the arrest of DNA replication. This finding suggests a role of Mre11 and RadA in the signaling and/or the repair of stalled replication forks.

Gamma irradiation did not lead to dramatic variations of Rad50, Mre11, HerA or NurA total levels. However, the slight increase of HerA at 7 h and 24 h of post-irradiation growth, the persistence of higher levels of both Rad50 and Mre11 at 24 h, and the physical interaction between Rad50, Mre11 and the HerA helicase support the involvement of the three proteins in DNA repair and suggest fine regulations of this pathway. No protein interaction was found with the NurA nuclease. Either this nuclease exhibits no or weak protein interactions with Rad50, Mre11 and/or HerA, or a competition for binding site accessibility prevents the recovery of NurA in our immunoprecipitation assays since the antibodies were raised against protein peptides. None of these proteins appears to display tight interactions with the RadA recombinase since, in that case, immunoprecipitation assays performed with antibodies raised against the whole RadA protein did not allow the recovery of any of the other proteins (not shown).

To date, DNA helicases that have been shown to functionally interact with the Rad50-Mre11 complex correspond to eukaryotic RecQ-like enzymes involved in checkpoint activation and in the resolution of DNA structure intermediates in the course of DNA recombination/repair processes [34,35]. However, it has been recently shown in *Xenopus laevis*, that the WRN (Werner's syndrome) RecQ-like helicase also acts at the initiation step of homologous recombination in the unwinding of broken DNA ends [36]. Archaea lack any RecQ homolog but might harbor several functional analogs that could play specific or overlapping functions as in eucarya. Indeed, in hyperthermophilic archaea, two helicases acting in the repair of stalled replication forks have been characterized, the Hjm/Hel308a helicase specific for branched DNA species [37-39], and Hef, a composite helicase/endonuclease protein [40,41]. The HerA helicase might also correspond to one of these RecQ analogs and could be primarily involved at the initiation step of homologous recombination in concert with the Rad50-Mre11 complex and the NurA nuclease. Intriguingly, we found that the DNA-bound fraction of HerA progressively increases along culture growth in both control and irradiated cells. Recently, Lundgren and Bernander analyzed cell-cycle genes expression in *S. acidocaldarius *[42]. Analysis of these data shows that *herA *expression is induced at first in S phase as it is the case for several DNA repair genes such as *radA*, while a second induction of *herA *is observed at the S/G2 transition. These findings suggest that in addition to its functions in DNA repair, HerA could be involved in chromosome segregation processes. We previously showed that HerA is the prototype of a novel helicase family distantly related to a NTPases family involved in DNA transport processes such as TrwB implicated in DNA conjugation, VirD4 implicated in DNA translocation from bacteria to plant cells or FtsK involved in DNA pumping during bacterial cell division [23]. As no true FtsK homolog is found in archaea, Iyer *et al*. have thus suggested that HerA could be the FtsK counterpart and could act as a DNA translocase in the course of chromosome segregation [43]. Considering the helicase activities associated with HerA [23], *herA *cell-cycle expression [42], and our present data, another hypothesis on the potential role of HerA in chromosome segregation processes could be that this helicase helps the correct segregation of newly replicated chromosomes as previously shown for the *S. cerevisiae *RecQ-like helicase Sgs1 [44].

## Conclusion

The Rad50 and Mre11 proteins have been conserved throughout the course of evolution. Our results provide the first *in vivo *data supporting the implication of the Mre11 protein in DNA repair processes in the Archaea and showing its physical interaction with Rad50 and the archaeal-specific HerA bipolar helicase. Moreover, they suggest that each protein might play several roles. Our current *in vitro *studies on the specificities and functional interactions between these proteins, the NurA nuclease and the RadA recombinase, combined to genetic analyses, should help define the roles and the mechanisms of action of these key proteins.

## Methods

### Sulfolobus acidocaldarius growth and irradiation

*S. acidocaldarius *DSM 639 cells were grown to early exponential phase at 78°C in Brock's medium pH 3 supplemented with 0.1% tryptone and 0.2% D-arabinose (generation time 6 h) [45]. The cultures were concentrated to 10^10 ^cells/ml and were irradiated on ice at a rate of 56.6 Gy/min using a ^137^Cs gamma ray source (Institute Curie, Orsay, France). Irradiations performed under limited-aerobic conditions were realized by the substitution of air for N_2_. For each irradiation dose (250, 500, 750, 1000, 1500 and 2000 Gy), survival rates were determined from three independent experiments by cells plating as described previously [46]. Mock cells were submitted to the same procedure without irradiation.

### Chromosomal DNA analysis by Pulsed Field Gel Electrophoresis (PFGE)

*S. acidocaldarius *cells irradiated at 1000 Gy under limited-aerobic conditions were re-inoculated into fresh medium at a concentration of 1.25 × 10^8 ^cells/ml and further grown up to the stationary phase (3.5 × 10^8 ^cells/ml). Aliquots were harvested by centrifugation after 0, 3, 5, 7 and 24 h of further growth, and cells were washed two times in TEN buffer (50 mM Tris-HCl pH 8, 50 mM EDTA pH 8, 100 mM NaCl), embedded in agarose plugs to a concentration of 5 × 10^9 ^cells/ml and lysed in NDS buffer (0.5 M EDTA, 50 mM Tris-HCl pH 9.5, 2% laurylsarkosine and 2 mg/ml Proteinase K) overnight at 37°C. Plugs were washed 3 times in NDS buffer pH 8. Chromosomal DNA from 10^8 ^cells was analyzed on a 1 % agarose gel in 0.5 × TAE buffer by PFGE (6 V/cm, 120° angle, 1s-30s, 18 h, CHEF-MAPPER Biorad) and visualized by ethidium bromide staining.

### Cell fractionation and protein analysis by Western blotting

Concomitantly to the sample collection for DNA analyses, aliquots (10^10 ^cells) of control and irradiated cells were harvested by centrifugation at 0, 3, 7 and 24 h of post-treatment growth, washed with 20 mM HEPES pH 7.5/1 M sorbitol buffer and were gently disrupted and fractionated by re-suspension in extraction buffer (50 mM Tris-HCl pH 7, 15 mM MgCl_2_, 50 mM NaCl, 1 mM DTT, 0.4 M sorbitol, 1 mM PMSF and Protease Inhibitor Cocktail Sigma) and by the addition of 0.5 % Triton X-100 (cells final concentration in lysis buffer 10^10 ^cells/ml). For experiments shown in figures 2b and 2c, an additional cell aliquot was harvested at time 3 and was incubated for 1 h at 37°C with 20 units DNase I (Roche) prior to cell fractionation. After 20 min on ice, equal volumes were collected for total and fractionated extracts preparation. Soluble (supernatant) and chromosomal DNA-enriched fractions (pellet) were recovered by centrifugation at 14000 g for 10 min at 4°C, and pellet fractions were washed once with extraction buffer and dissolved in an equal volume of extraction buffer. 30 ul of each extract were run on SDS-gels and submitted to Western blotting using specific polyclonal antisera and alkaline phosphatase conjugated anti-IgGs. Antisera against Rad50, Mre11, HerA and NurA were raised against protein peptides and antisera against RadA and DNA ligase were raised against the whole proteins. All antisera reacted with the corresponding purified recombinant protein and recognized one major band of expected molecular weight in total extracts of *S. acidocaldarius*. Quantification analyses of protein bands detected by Western blot were performed using the ImageJ 1.38 software [47].

### Immunoprecipitation assays

Immunoprecipitations were performed on *S. acidocaldarius *extracts (0.5 ml/5 × 10^8 ^cells) from exponentially growing cultures obtained as described above or on a mixture (0.3 ml) of purified recombinant proteins containing 60 nM of Rad50, Mre11, and NurA and 300 nM of HerA in IP buffer (20 mM Hepes pH 7.5, 100 mM NaCl, 15 mM MgCl_2 _and 5 mM beta-mercaptoethanol). NurA and HerA proteins were purified as described in Constantinesco *et al*. [22,23], and Rad50 and Mre11 proteins were purified by chromatography through Ni-NTA (Quiagen) and gel filtration though Sephadex 200 (Amersham Biosciences, purification details will be published elsewhere). Samples were incubated with 10 μl of pre-immune or anti-Rad50 purified IgGs for 3 h at 4°C with gentle shaking. Immunocomplexes were collected by adding 50 μl of BSA-saturated proteinA-sepharose beads (Amersham Biosciences) with gentle shaking for 2 h at 4°C, followed by extensive washes with IP buffer. Proteins were eluted by boiling treatment in 2 × electrophoresis sample buffer, separated by SDS-PAGE and identified by immunoblotting.

## Authors' contributions

AQ carried out cell cultures and irradiations, DNA analyses by PFGE, Western blots and immunoprecipitations assays and helped to draft the manuscript. FC carried out recombinant proteins purifications, participated in the design of antibodies, and helped to draft the manuscript. MW provided RadA antibodies and participated in the critical revision of the manuscript. PF participated in the critical revision of the manuscript. CE conceived the study, participated in the design of antibodies and in protein purifications, and drafted the manuscript. All authors read and approved the final manuscript.
